# City Noises Versus Health and Longevity

**Published:** 1897-07

**Authors:** W. F. McNutt

**Affiliations:** Professor Practice of Medicine University of California


### SELECTIONS.

### City Noises Versus Health and Longevity.

BY W. F. MCNUTT, M.D., M.R.C.S., ED , PROFESSOR PRACTICE OF MEDICINE UNIVERSITY OF CALIFORNIA.

(Read before the Medical Society of the State of California, April, 1897.)

That rest,—undisturbed repose,—is essential to health, and therefore conducive to longevity, is a sanitary axiom which will not be questioned. It is also axiomatic undisturbed repose, under the most favorable city conditions, is extremely hard to obtain.

Every organ in the body is so constituted that over-stimulation will so disturb its physiological action, that sooner or later a pathological condition,—a degenerative process,—is the result. A quarter of a century's practice of medicine in a city teaches a physician that there are many deteoriative influences at work, sapping the health of the denizens of a great city, and that not the least of these nerve strains,—these wretched miseries,—these many deteriorating factors,—is noise. True, we have no apparatus for measuring the exact number of years that the average city life is shortened, or the inroads that are made upon an individual's health by the useless and unnecessary noises of a city. Other things being equal, the man who has his place of business and his residence on smooth, noiseless street, can work more hours per day, and will live longer than the man who is subject all day and all night to the noise and rattle of a rough stone street. Byrne, in Highway Construction, says "Rough surface pavements are noisy under traffic, and insufferable to nervous invalids, and much nervous sickness is attributable to them. To all persons interested in nervous invalids, this damage from noisy pavements is rated as being far greater than would be the cost of substituting the best quality of noisless pavements." Much of the noise of a city must be endured as a necessary evil; very much is unnecessary and should be prevented. It should be as much a part of the duties of the Health Department of a city to prevent unnecessary noise, as to prevent any other unsanitary condition, in the interests of the health of the citizen. Many of the nuisances that are at present receiving attention from Boards of Health are much less conducive to the misery and ill health of the average citizen than is that of noise. The Health Department is in duty bound to promote the comfort and well being of the citizen, and to minimize his discomforts.

From a purely esthetic standpoint many of the city noises should be abolished, but from a hygienic and physiological standpoint they are a source of disease and misery, and hence maintained at great economic loss to the municipality. Not long since while visiting a nervous degenerate, a poor broken-down hysteric woman, and while waiting for the husband's return to tell him that his wife could neither regain her health, nor maintain it in her present location, that she was being slowly and cruelly tortured to death by the noises of her surroundings, he came in from a meeting of the Society for Prevention of Cruelty to Animals, of which he was an enthusiastic member. It would be natural to suppose that such a persons humane instincts might be enlisted against the noisemakers in the interest of a hysteric wife, whose nervous system had given way under the constant noise shocks, the unrest of her surroundings. Such is the inconsistency of human nature, however, that he could not resist every and all forms of noise.

Every physician has recognized the baneful effects of noise upon many a patient. How impossible it seems to secure the necessary absolute rest from noise for his or her recovery, how every passing team on the hard stones lessens the vital resistance and makes recovery a little less certain, and death a little more sure! Who of us has not seen a life saved by a few loads of tan bark spread on the stones under the patient's window, and yet we patiently submit to the stone street. And worse, we often place our patients in private houses or hospitals that are located where the noisemakers are the most persistent, and where rest is impossible. We boast of our modern civilization, our progressive age, the many additions to our comfort, and we take no note of our unnecessary discomforts. It is not enough that we must endure the cable-car rattle and the hum of the trolly-car twenty hours out of twenty-four, but the other four hours of the night must be made hideous by a gang of track repairers. Were a citizen to object to the night gang on the grounds of disturbing his rest, he would probably be considered insane. One of the most curious phases of our modern social fabric is the submission of the people of a city to the unnecessary noisemakers. Let one seat himself at the window of a house on a busy car-line street and hear the eternal noise of the car, the constant ringing of the bells, the shrieking and bellowing of the hucksters of all kinds and conditions, the constant rattle, rattle of the teams all day long and away into the night. And that night is made hideous by a thousand unnecessary and necessary disturbances,—steam whistles startling the sleeper, church and factory bells ringing at unearthly morning hours, milk, baker and butcher wagons, with rounders and loafers. One would be surprired to notice how many of these street noises are unnecessary, and still more surprised that citizens will continue to endure them. The rattle and clatter and jarring on the stone block and cobble pavements, and unnecessary street hawkers, and hand-organ fakirs, and howling dogs, and bellowing cows are particularly exasperating, because they are absolutely unnecessary. All unnecessary night noises should be suppressed. The night should be sacred, at least a few hours of each night should be consecrated to the muscle and brain workers and the sick; sleep is nature's sweet restorer, brain work and muscle work must be done; rest is necessary in order that the product of either be efficient. Undisturbed rest is nature's sovereign prophylatic against disease; without rest disease will "invade the chastest temperance" and the most robust of constitutions. Why, for instance, should a whole neighborhood of tired citizens have their sleep disturbed, or it be made quite impossible to sleep at all, because some inhuman monster chooses to keep open a dive where a dozen degraded wretches make night hideous? What are municipal legislative bodies for? What better use can a police officer, or a health inspector, make of his time than in protecting the community from disease? If medical men, through sanitary associations, medical societies and boards of health, will but educate the people to a realizing sense of noise as a cause of disease, legislation for the suppression of noisemakers would be demanded. So long as the voter is uneducated, is ignorant of the causes of disease, the ordinary municipal officer will neither acquire sanitary knowledge nor enforce the observance of sanitary laws. When not controlled by the tyranny of mighty corporations, he draws his inspiration from his constituency and hopes by so doing to be promoted or at least re-elected. Let us educate his constituency. The sanitarian and reformer must take courage, they must have compassion on and patience with the multitude. Scientific knowledge spreads slowly, but it surely spreads; the multitude will some day demand of its servants, the municipal officers, protection from vice and disease. City life will be made more tolerable, the life span will be prolonged. The necessity for rest will be better appreciated, saloons will all be closed at midnight, rioters will be fewer, policemen will be instructed to suppress unnecessary noises. When stone blocks and cobbles give way to smooth streets, and bakers' and butchers' and milk wagons have rubber tires, they will cease to be the night fiends that they are at present. The sick and the weary will not have their needful necessary sleep disturbed by steam whistles and church bells. Science will finally triumph, intelligence will prevail over ignorance, license will cease to be recognized as liberty, the fittest must survive.—*Ex.*

